# Apelin affects the mouse aging urinary peptidome with minimal effects on kidney

**DOI:** 10.1038/s41598-019-47109-4

**Published:** 2019-07-23

**Authors:** Claire Vinel, Joost P. Schanstra, Franck Boizard, Ophélie Péreira, Johanna Auriau, Alizée Dortignac, Benjamin Breuil, Guylène Feuillet, Esther Nkuipou-Kenfack, Petra Zürbig, Philippe Valet, Jean-Loup Bascands, Cédric Dray, Colette Denis

**Affiliations:** 1 Institut National de la Santé et de la Recherche Médicale (INSERM), U1048, Institut of Cardiovascular and Metabolic Disease, Toulouse, France; 2 Université Toulouse III Paul-Sabatier, Toulouse, France; 3 Mosaiques diagnostics GmbH, Hannover, Germany; 4 Institut National de la Sante et de la Recherche Medicale (INSERM), U1188 - Université de La Réunion, Saint-Denis, France

**Keywords:** Kidney, Ageing

## Abstract

Kidney function is altered by age together with a declined filtration capacity of 5–10% per decade after 35 years. Renal aging shares many characteristics with chronic kidney disease. Plasma levels of the bioactive peptide apelin also decline with age and apelin has been shown to be protective in chronic kidney disease. Therefore we evaluated whether apelin could also improve aging-induced renal lesions and function in mice. Since urine is for the major part composed of proteins and peptides originating from the kidney, we first studied apelin-induced changes, in the aging urinary peptidome. Despite the recently published age-associated plasma decrease of apelin, expression of the peptide and its receptor was increased in the kidneys of 24 months old mice. Twenty-eight days treatment with apelin significantly modified the urinary peptidome of 3 and 24 months old mice towards a signature suggesting more advanced age at 3 months, and a younger age at 24 months. The latter was accompanied by a decreased staining of collagen (Sirius red staining) in 24 months old apelin-treated mice, without changing aging-induced glomerular hypertrophy. In addition, apelin was without effect on aging-induced renal autophagy, apoptosis, inflammation and reduced renal function. In conclusion, treatment of aged mice with apelin had a limited effect on kidney lesions although modifying the urinary peptidome towards a younger signature. This supports evidence of apelin inducing more general beneficial effects on other aging organs, muscles in particular, as recently shown for sarcopenia, markers of which end up via the glomerular filtration in urine.

## Introduction

With the increase in life expectancy in Western countries, maintaining elderly people in good health has become a crucial challenge. Aging is a complex systemic process leading to the gradual decline of physiological functions. Among them, kidney function is often altered with age. The glomerular filtration rate is reported to decline by 5–10% per decade after 35 years^1^. This is the result of a loss of functioning nephrons by a focal and global glomerulosclerosis process, occurring in healthy aging, which is not associated to compensatory hyperfiltration of the remaining nephrons^2^. Tubular atrophy in association with interstitial fibrosis and microvascular rarefaction is also often described in aging kidneys. These combined changes lead to a reduced capacity of the kidney to maintain sodium balance, to concentrate or dilute properly urine and to counteract metabolic acidosis^3,4^. The decline progresses slowly in healthy people. Nevertheless, aging decreases the recovery capacity of kidney after acute kidney injury^1^. In association with other risk factors (hypertension, diabetes), the prevalence of chronic kidney disease (CKD) rises sharply with age and age is a key predictor of CKD^5^. Furthermore, analysis of human urinary proteome suggested important similarity between the aging kidney and CKD^6^.

The bioactive peptide apelin was recently described as a peptide whose production declines with age and which is able to reverse age-associated sarcopenia^7^ and to improve mammalian healthspan^8^. Apelin (Apln) exists under different forms, from 13 to 36 amino acids, all-originating from preproapelin, a precursor of 77 amino acids. The apelin gene is expressed in many tissues, such as blood vessels, stomach, muscle, adipose tissue, heart, lungs, and different brain areas^9^. In most cases, APJ, the apelin receptor, is also present in the tissues expressing apelin. In the kidney, the presence of apelin was first described in the human collecting duct^10^. mRNA for preproapelin and the APJ receptor were subsequently detected in human kidney extracts^11^. In mice, APJ mRNA was found throughout the renal cortex and medulla^12^. The presence of the receptor protein was confirmed in these areas^13^. In the rat kidney, APJ receptor mRNA was detected in glomerular arterioles and to a lesser extent all along the tubule, and it was observed that apelin was able to overcome Angiotensin II mediated vasoconstriction^14^. In the collecting ducts, apelin counteracted the antidiuretic effect induced by vasopressin in a rat model^15^.

Protective effects of apelin have been demonstrated in CKD. Indeed, it has been shown that apelin reduced interstitial fibrosis in kidneys of mice following unilateral ureteral obstruction, by attenuating epithelial-mesenchymal transition^16^. This protective effect was also demonstrated in rat kidneys following ischemia-reperfusion^17^ and in several models of diabetic nephropathy^18–20^.

As renal aging shares many characteristics with CKD and since apelin treatment significantly restored muscle function in aged mice^7^, we tested the hypothesis whether apelin could also improve upon aging-induced renal lesions and function.

## Results

### Urinary proteome analysis

**Figure 1:**
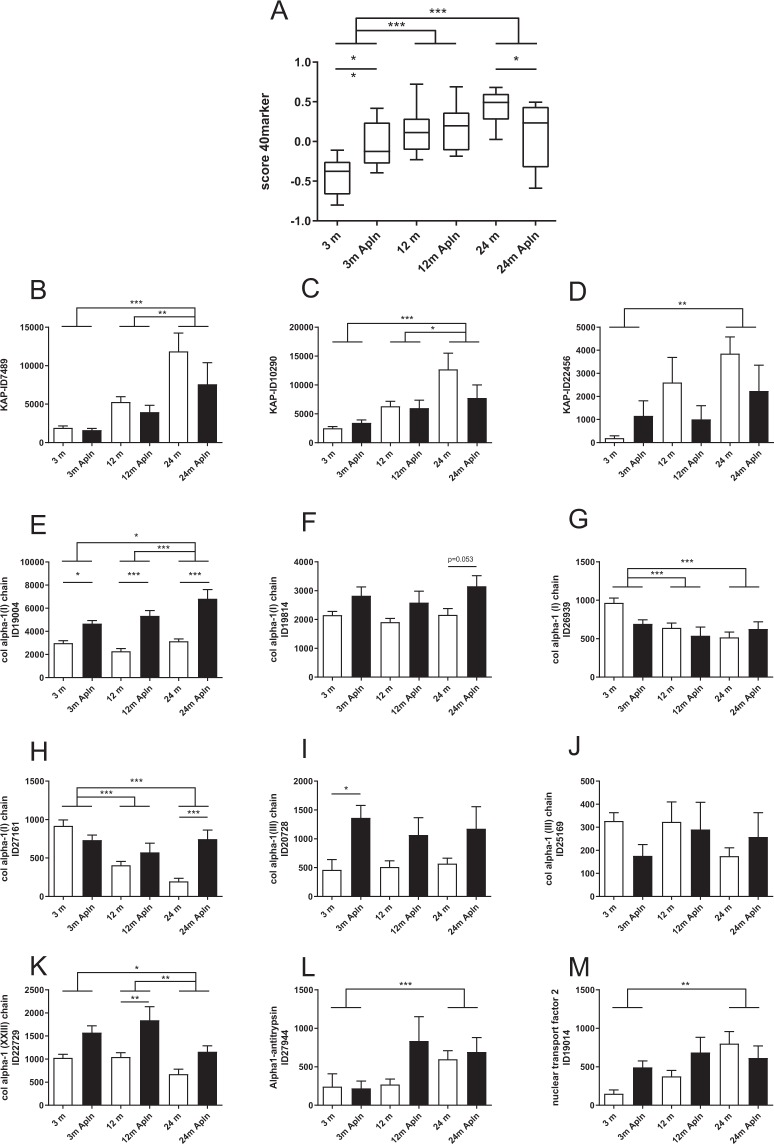
Urinary proteome analysis in control and Apln-treated mice at different ages. (**A**) Scoring of different ages treated with or without apelin based on a model of 40 urinary peptides associated to aging in mice. (**B**–**M**) Abundance of 12 sequenced peptide fragments. (**B**) Peptide ID 7489 from Kidney androgen-regulated protein; (**C**) peptide ID 10290 from Kidney androgen-regulated protein; (**D**) peptide ID 22456 from Kidney androgen-regulated protein; (**E**) peptide ID19004 from Collagen α-1(I) chain; (**F**) peptide ID198144 from Collagen α-1(I) chain; (**G**) peptide ID26939 from Collagen α-1(I) chain; (**H**) peptide 27161 from Collagen α-1(I) chain; (**I**) peptide 20728 from Collagen α-1(III) chain; (**J**) peptide 25169 from Collagen α-1(III) chain; (**K**) peptide 22729 from Collagen α-1(XXIII) chain; (**L**) peptide ID 27944 from α-1-antitrypsin 1–4; (**M**) peptide ID 19014 from Nuclear transport factor 2. All values are mean ± sem. Statistical analysis used two-ways Anova and Tukey’s post test. *P < 0.05, **P < 0.01 or ***P < 0.001.

The human urinary peptidome is modified significantly with age and these modifications are strongly linked to CKD^6^. Therefore the analysis of the urinary peptidome would allow to non-invasively study the effect of apelin on aging-induced CKD. With this aim, we analyzed the urinary proteome of mice at 3, 12 and 24 months, treated daily with or without apelin-13 (0.5 µmol/kg/d, ip) or PBS for 28 days. To generate a reference mouse urinary peptidome of mouse aging we used data from a previous study^21^ (described in the Methods section). This resulted in the development of a support vector machine (SVM) mathematical model based on 40 urinary mouse peptides associated with age. Applying this SVM model on control mice (i.e. not treated with apelin) aged 3, 12 and 24 months clearly discriminated between the 3 ages of the untreated mice (Fig. 1A) thereby validating the SVM urinary peptide model. Among the 40 peptides used in this model, for 12 the actual amino-acid sequence could be obtained (Table 1). They represent fragments of kidney androgen-related protein, different collagen proteins, α-1antitrypsin and nuclear transport factor 2. The relative abundance of some of these peptides was significantly increased with age (Fig. 1B–M): Kidney androgen-regulated protein, α-1-antitrypsin 1–4 and Nuclear transport factor 2 fragments. By contrast, some collagen fragments from type I fibrillary collagen and type XXIII transmembranous collagen were decreased. The abundance of other peptides originating from collagen I or III was unchanged. When the mice were treated for 28 days with apelin, the scores of the 40-peptide model were modified, leading to significantly different scores at 3 and 24 months (Fig. 1A). In 3 months old mice, apelin induced an increase of the score, as if the animals were older than controls. Interestingly, when 24 months old animals were treated with apelin, the opposite was observed since the score decreased to reach value close to 12 months old mice and suggesting rejuvenation. Among the 12 sequenced peptides, apelin significantly increased the relative abundance of 2 collagen type I fragments.

**Table 1:** Sequence of 12 out of the 40 urinary peptides associated to mouse aging.

Peptide ID	Sequence	Prot_accession	Protein name	Start AA	Stop AA
27944	EEHTQSPIFVGKVVDPTHK	Q00897	Alpha-1-antitrypsin 1–4	395	413
19004	GQpGAkGEpGDTGVKGD	P11087	Collagen alpha-1(I) chain	810	826
19814	GLpGPAGPpGEAGKpGEQ	P11087	Collagen alpha-1(I) chain	633	650
26939	GQPGAKGEpGDTGVKGDAGPpGP	P11087	Collagen alpha-1(I) chain	810	832
27161	GQpGAKGEpGDTGVKGDAGPpGP	P11087	Collagen alpha-1(I) chain	810	832
20728	GmPGSpGGpGNDGKpGpPG	P08121	Collagen alpha-1(III) chain	536	554
25169	QGIpGTGGPpGENGKpGEpGP	P08121	Collagen alpha-1(III) chain	640	660
22729	pGpAGpKGETGEmGLSGLP	Q8K4G2	Collagen alpha-1(XXIII) chain	357	375
7489	VSINKELQNS	P61110	Kidney androgen-regulated protein	25	34
10290	VSINKELQNSI	P61110	Kidney androgen-regulated protein	25	35
22456	SINKELQNSIIDLLNS	P61110	Kidney androgen-regulated protein	26	41
19014	VVGQLKADEDPIMGF	P61971	Nuclear transport factor 2	85	99

Since it is believed that peptides present in urine originate mainly from kidneys^22^, we therefore tried to associate the apelin-induced changes in the urinary peptidome to modifications on kidney structure and function.

### Structural modifications and fibrotic process in kidneys

**Figure 2:**
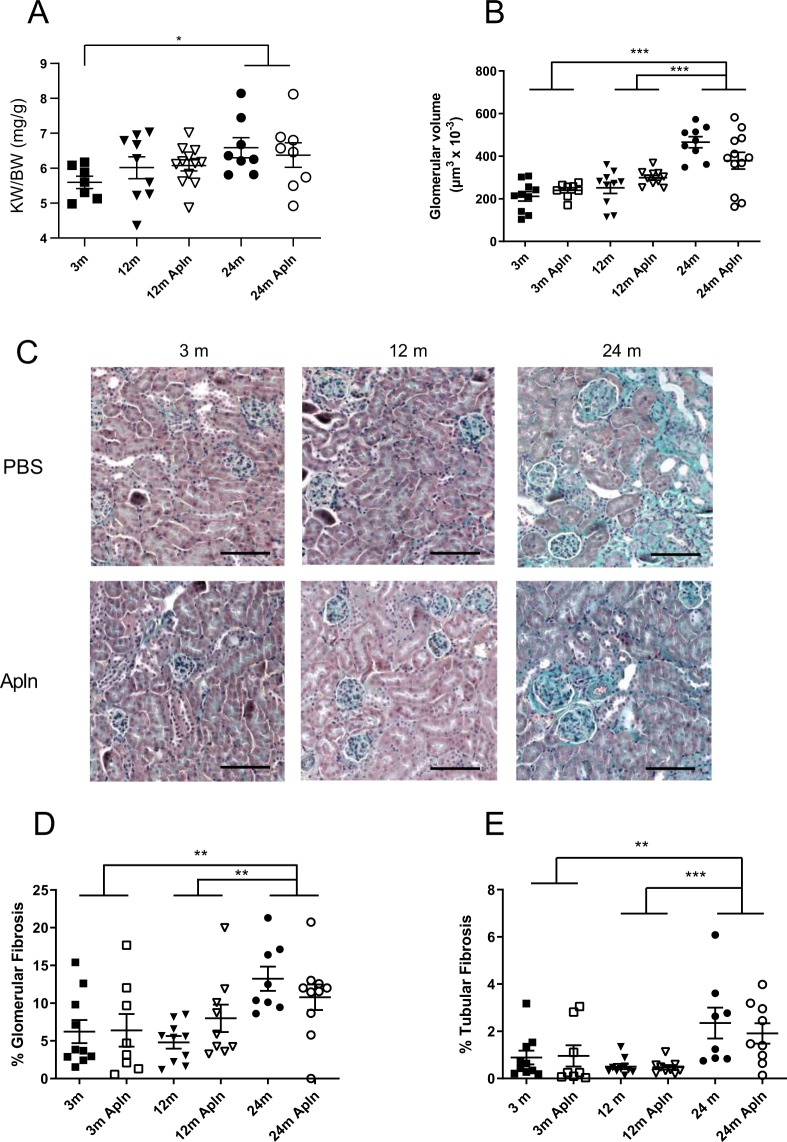
Structural modifications and fibrosis in kidneys of control and Apln-treated mice at different ages. (**A**) Kidney weight/Body weight ratio; (**B**) glomerular volume calculated from Masson’s trichrome image analysis. (**C**) Representative Masson’s trichrome stained images of mice treated for one month with PBS or with Apln, bar represents 100 μm; (**D**) % total glomerular area occupied by fibrosis and (**E**) % total tubular area occupied by fibrosis, from Masson’s trichrome images. All values are mean ± sem. Statistical analysis used two-ways Anova and Tukey’s post test. *P < 0.05, **P < 0.01 or ***P < 0.001.

Aging induced noticeable structural modifications in kidneys. First, the ratio KW/BW was increased by 19% between 3 and 24 months (Fig. 2A). An ultrastructural study of kidneys in Masson’s trichrome stained slides allowed us to determine the mean glomerular area and to calculate the glomerular volume, as shown in Fig. 1B. A significant glomerular hypertrophy was observed with age (24 m vs 3 m and 12 m) since the glomerular volume was more than doubled between 3 and 24 months of age. However apelin treatment did not significantly modify these parameters.

**Figure 3:**
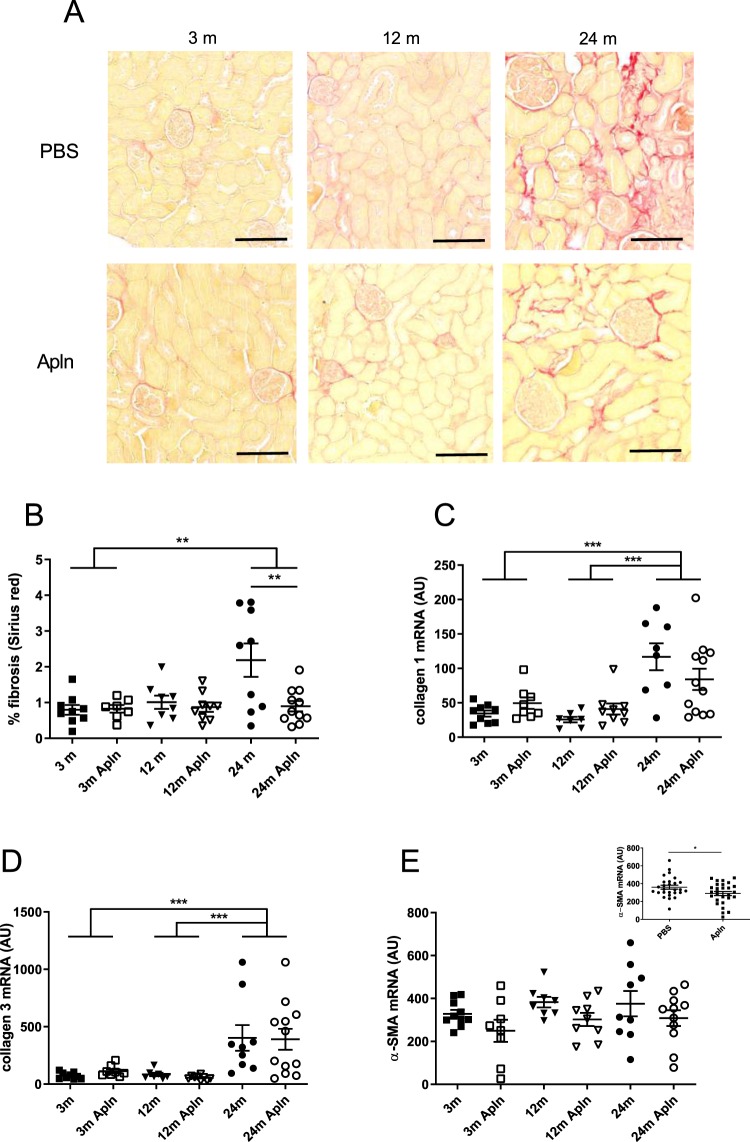
Analysis of fibrosis, collagen and a-SMA expression in kidneys of control and Apln-treated mice at different ages. (**A**) Representative Sirius red stained images, of mice treated for one month with PBS or with Apln, bar represents 100 μm; (**B**) % total cortex area occupied by fibrosis, from Sirius red stained images. Collagen 1 (**C**), collagen 3 (**D**) and α-SMA (**E**) mRNA expression in mice kidneys. Inset in E represents values of PBS and Apln treated animals independently of age. All values are mean ± sem. Statistical analysis used two-ways Anova and Tukey’s post test. *P < 0.05, **P < 0.01 or ***P < 0.001.

This hypertrophy was accompanied by a clear increase in glomerular fibrosis (Fig. 2C,D), more pronounced than the tubulo-interstitial fibrosis (Fig. 2C–E). At 24 months, fibrosis reached 13% of the glomerular area vs 2% of the tubular area. No significant effect of the apelin treatment was observed in both cases. Sirius Red staining also demonstrated an increase in collagen deposition in the renal cortex of old mice (Fig. 3A,B). In 24 months old mice, apelin treatment significantly decreased the Sirius red staining, suggesting reduced collagen deposition following treatment. In accordance with this increase in fibrosis with age, collagen I and III mRNA abundance was increased in kidneys of 24 months old animals (Fig. 3C,D). However, there was no significant effect of apelin treatment on the expression of the collagen mRNA suggesting that this effect might be post-translational. Renal α-SMA mRNA abundance, a marker of myofibroblasts, did not change with age and was not modified by apelin at the different ages (Fig. 3E). However, α-SMA mRNA abundance was the only parameter that was lower after apelin treatment independent of age (Fig. 3E, inset).

### Expression of inflammation, autophagy and apoptosis markers

**Figure 4:**
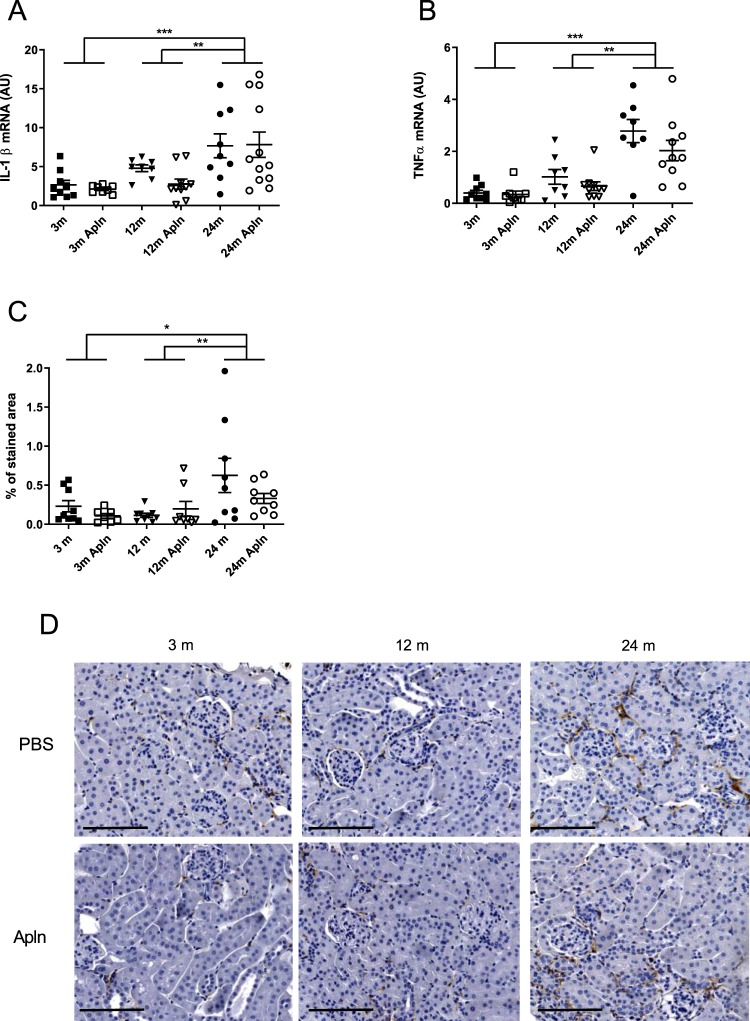
Analysis of inflammation markers in kidneys of control and Apln-treated mice at different ages. IL1-β (**A**) and TNFα (**B**) mRNA expression in mice kidneys. (**C**) % of cortex area occupied by F4/80 staining (macrophage marker). (**D**) Representative F4/80 stained images in kidneys of mice treated with PBS or Apln, bar represents 100 μm. All values are mean ± sem. Statistical analysis used two-ways Anova and Tukey’s post test. *P < 0.05, **P < 0.01 or ***P < 0.001.

**Figure 5:**
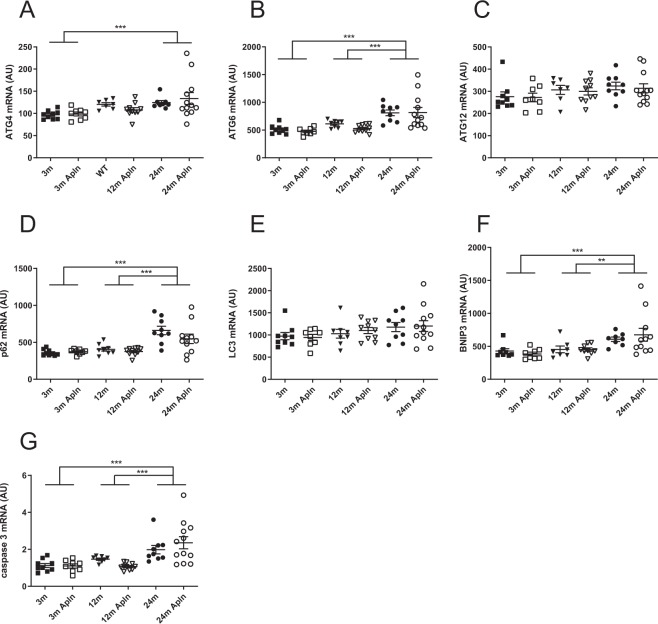
Analysis of autophagia and apoptosis markers in kidneys of control and Apln-treated mice at different ages. Expression of mRNA for ATG4 (**A**), ATG6 (**B**), ATG12 (**C**), p62 (**D**), LC3 (**E**), Bnip3 (**F**), caspase 3 (**G**) in kidneys. All values are mean ± sem. Statistical analysis used two-ways Anova and Tukey’s post test. *P < 0.05, **P < 0.01 or ***P < 0.001.

The inflammatory status of the kidney was evaluated by several means: cytokines IL-1β and TNF-α mRNA expression and staining of kidney slides with F4/80 antibody, a marker of macrophages. As shown in Fig. 4, inflammation was significantly enhanced at 24 months, compared to 3 or 12 months. Moreover, the expression of several genes involved in autophagia and apoptosis was also investigated in kidneys from mice at different ages (Fig. 5). The mRNA expression of several autophagy markers, ATG4, ATG6 (beclin-1) and p62, the mitophagy marker Bnip3 and the apoptosis marker caspase 3 was increased in 24 months old animals compared to younger mice. By contrast, mRNA expression of ATG12 and LC3 was not modified with aging. However, apelin did not modify the expression of any of these markers.

### Albuminuria and apelinergic system in kidneys

**Figure 6:**
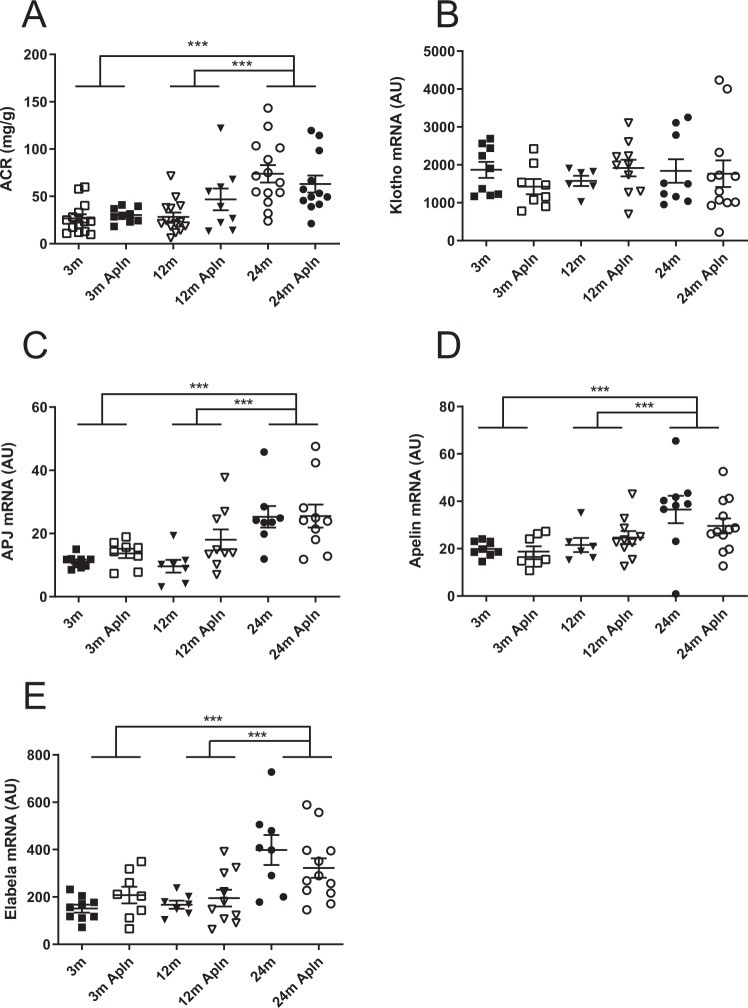
Analysis of albuminuria, Klotho and apelinergic system expression in kidneys of control and Apln-treated mice at different ages. (**A**) Urinary albumin on creatinine ratio (mg/g); (**B**) Klotho mRNA expression; (**C**) APJ (Apelin receptor) mRNA expression; (**D**) Apelin mRNA expression; (**E**) Elabela mRNA expression in kidneys. All values are mean ± sem. Statistical analysis used two-ways Anova and Tukey’s post test. *P < 0.05, **P < 0.01 or ***P < 0.001.

The structural alterations were accompanied by several functional modifications as shown in Fig. 6. First, at 24 months, the excretion of albumin in urine was enhanced, with a three times increase in the albumin/creatinine ratio which was not modified by apelin treatment. We also investigated renal mRNA expression of Klotho, a transmembrane protein whose lack is associated with premature aging. No change in Klotho mRNA abundance was noticed, regardless of age or treatment. We then considered the renal apelinergic system by investigating APJ, Apelin and Elabela mRNA expression. Elabela is another APJ ligand which is almost exclusively synthesized in kidney. The apelin receptor APJ mRNA was more abundant in kidneys of 24 months old mice compared to 3 or 12 months old animals, in contrast to the decreased expression found in muscle^7^. We also noticed a significant rise in apelin and Elabela mRNA in kidneys from 24 months old mice. Apelin treatment did not induce any significant modification of these parameters.

## Discussion

Although apelin has shown beneficial effects in a number of models of kidney disease, the current study provides evidence that apelin has minimal effects on aging-induced kidney lesions in mice.

Our results confirm similarities between the aging process and the development of CKD^4^, in particular the rise of albuminuria, histological changes such as glomerular hypertrophy and tubulo-interstitial fibrosis. The decrease of collagen fragments in urine represents also a strong similarity between renal aging and CKD in humans, as already emphasized by Zurbig *et al*.^6^. However, lesions are gradual with age in contrast to more progressive injuries seen in pathological cases. Nevertheless, these changes predispose the kidney of healthy aging people to become more susceptible to other diseases such as hypertension, diabetes and CKD^5^.

When the mice were treated with apelin, very few modifications were observed in the aging-induced alterations in the kidney, distinguishing in this respect aging from CKD. The same is true in case of apelin deficiency since we did not see any significant changes in 12 months old apelin KO mice compared to WT using the peptidome score, glomerular volume, collagen I and III mRNA expression (not shown). This result is in accordance with Rai *et al*.^8^ who demonstrated that apelin-deficient mice did not exhibit renal alterations in contrast to APJ KO mice. The only clear effect obtained after apelin supplementation was a reduction of renal fibrosis in mice of advanced age (24 weeks) after apelin treatment as estimated by Sirius red staining. Nevertheless, no significant modifications of other *in-situ* fibrosis markers were noticed after apelin treatment. The discrepancy between Masson’s and Sirius staining results may be explained by the fact that Masson’s trichrome detects all forms of collagen and probably more matrix components than does Sirius red which is more specific for collagen I and III^23^. Although the collagen deposition as assessed by Sirius red was lower in 24 months old animals treated with apelin, the expression of collagen I and III mRNA was not modified. This suggests that collagen degradation rather than synthesis was modified by apelin treatment. This is supported by the observation of significantly increased urinary collagen I fragments after apelin treatment in 24 old mice. This minimal effect of apelin in aged mice is opposed to the beneficial effects of this peptide observed in CKD models. In that respect, apelin-13 decreased proteinuria, histological alterations and suppressed inflammation in the Akita mouse, a model of diabetic nephropathy^20^ and also delayed the progression of nephropathy in Ove26 mice, another model of type 1 diabetes^19^. In a model of unilateral ureteral obstruction, administration of apelin significantly reduced interstitial fibrosis and TGFβ expression^24^. In rats exposed to renal ischemia/reperfusion injury apelin-13 inhibited the elevation of inflammatory factors, the morphological alterations in kidney and proteinuria^25^. This protective effect was confirmed and linked to increased antioxidant enzymes activities by Bircan *et al*.^17^. In accordance with these beneficial effects of apelin, in autosomal dominant polycystic kidney disease patients, a negative correlation between plasma apelin and TGFβ was found, while apelin was positively correlated to eGFR suggesting that although many similarities are observed between aging and CKD, the underlying mechanisms are different and do not present the same sensitivity to apelin treatment.

Although we did not observe evidence of modification of α−SMA mRNA in the kidneys upon aging, in contrast to what is known in CKD^26^, α−SMA mRNA abundance was the only parameter that was significantly lowered independently of age by apelin treatment. This supports a similar effect observed in the heart by Pchejetski *et al*.^27^, where an apelin pretreatment inhibited myocardial fibrotic remodeling and α-SMA expression after an aortic banding. The decreased expression of α-SMA with apelin was also documented in human proximal tubular epithelial cells in culture treated with TGFβ1^16^.

Although little effect of apelin treatment was observed in the kidney, increased mRNA expression of components of the apelinergic system: apelin, its receptor APJ and another APJ receptor agonist, elabela, was observed in the aging kidney. However, none of these were modified by apelin treatment. This result was expected since it has been shown that apelin treatment either increases or has no effect on apelin expression. On the other hand, Rai *et al*., observed a decrease of the apelin protein in renal tissues of 24 months old compared to 1-month-old mice^8^.This discrepancy may be explained by the age of the young mice (the comparator group) which is not the same in our study (3 months vs 1 month) and by the parameter studied (protein *vs* mRNA). Discrepant data were also observed for the APJ receptor since Rai *et al*. observed a decrease in APJ receptor mRNA abundance in the kidney with aging^8^. Better definition of the expression changes of the different components of the apelinergic system in the kidney during aging is thus warranted.

In conclusion, although apelin has minimal effects on aging-induced renal alterations in mice, the effect of apelin on the aging urinary peptidome is of interest. It may be due to the fact that peptides present in the urine are not exclusively of renal origin and about 30% is derived from plasma^22^. This suggests that apelin induces beneficial effects on other organs, muscles in particular, such as recently shown for sarcopenia, reflected by changes in the abundance of urinary peptides originating from these tissues^7^.

## Methods

### Animals

All experimental procedures were performed in accordance with institutional guidelines for animal studies and were approved by an ethics committee (US006/CREFRE, CEEA-122 INSERM). C57Bl6/J Wild-Type (WT) mice from 3 to 24 months-old were obtained from Janvier Laboratory (St-Berthevin, France). Mice were housed conventionally in a constant temperature (20–22 °C) and humidity (50–60%) animal room, with a 12/12 h light/dark cycle and free access to food and water. During the protocol, mice were excluded if they died prematurely or if they displayed tumors after necropsy. All mice were fed with normal diet. Urine was collected by placing the mice overnight in metabolic cage, 5 days before sacrifice. All mice were euthanatized in a fed state by cervical vertebra dislocation. Kidneys were removed and a portion of the renal cortex was snap-frozen in liquid nitrogen and stored at −80 °C for mRNA analysis. Another portion of kidney cortex was fixed in Carnoy’s solution for 24 h and subsequently embedded in paraffin for immuno-histological analysis.

### Apelin supplementation by chronic intra-peritoneal injection

3, 12 and 24 months-old C57Bl6/j mice were randomized in different groups according to their weight and were treated with 0.5 umol/kg/day of pyroglutaminated apelin-13 (Bachem, Switzerland) which has been previously shown to be the most simportant apelin isoform in blood^28^. The dose and frequency of apelin treatment have been determined in a previous study on muscle function in aged mice^7^. Age-matched control mice were PBS-injected. Investigators were blinded to the group allocation (injection of apelin or PBS) during the protocol and the associated-experiments. All mice were euthanatized 24 h after the last apelin or PBS injection.

### Urinary peptidome analysis

After thawing on ice, one hundred fifty µl of mouse urine sample was diluted with the same volume of a solution composed of 2 M urea, 10 mM NH4OH and 0.2% SDS. Subsequently, samples were ultrafiltered using a Centristat 20 kDa cut-off centrifugal filter device (Satorius, Göttingen, Germany) to eliminate high molecular weight molecules. The filtrate was desalted using a NAP5 gel filtration column (GE Healthcare Bio Sciences, Uppsala, Sweden) to remove urea and electrolytes, and thereby to decrease matrix effects. The sample was lyophilized in a Savant speedvac SVC100H connected to a Virtis 3 L Sentry freeze dryer (Fischer Scientific, Illkirch, France) and stored at 4 °C until use. Shortly before CE-MS analysis, the samples were re-suspended in 10 µL HPLC grade H2O.

CE-MS analysis was performed as previously described^29^. Briefly, CE-MS analyses were performed using a Beckman Coulter Proteome Lab PA800 capillary electrophoresis system (Beckman Coulter, Fullerton, USA) on-line coupled to a micrOTOF II MS (Bruker Daltonic, Bremen, Germany). The electro-ionization sprayer (Agilent Technologies, Palo Alto, CA, USA) was grounded, and the ion spray interface potential was set to −4.5 kV. Data acquisition and MS acquisition methods were automatically controlled by the CE via contact-close-relays. Spectra were accumulated every 3 s, over a range of m/z 350 to 3000. MosaiquesVisu software package was applied to deconvolve mass spectral ion peaks representing identical molecules at different charge states into single masses^30^. Migration time and ion signal intensity (amplitude) were normalized using internal polypeptide standards^31^. Each polypeptide present in the list was defined by its normalized migration time [min], molecular mass [kDa], and signal intensity detected. Using a Microsoft SQL database, all detected polypeptides were deposited, matched, and annotated in order to allow for further comparison between the groups. The criteria applied to considered a polypeptide identical was that within different samples the mass deviation was lower than 50 ppm for masses <4 kDa, 150 ppm for masses >6 kDa, and between 50–150 ppm for masses between 4–6 kDa. Acceptable migration time deviation was between 1 and 2.5 minutes.

### Development of a high dimensional urinary peptide model of mouse ageing

For the generation of a non-invasive peptide model associated with aging in mice, we compared the urinary peptides of 39 mice of a previous study^21^ at the age of 1 (n = 13), 12 (n = 13) and 21 (n = 13) months. Although this age span is not exactly the same as in the current study it allows to extract peptides associated to ageing. This comparison led to the identification of several significantly excreted peptides (adjusted (maxT step-down approach)^32^ Wilcoxon p value < 0.05). The comparison between young and middle age (n = 94 peptides with differential abundance), young and old age (n = 113 peptides), and middle and old age (n = 9 peptides) enabled to select 48 peptides present in at least 2 of the comparisons. These peptides were then included in a support vector machine (SVM) mathematical model using the proprietary software MosaCluster (version 1.7.0) that allows the classification of samples in the high dimensional data space^33,34^. MosaCluster calculates classification scores based on the amplitudes of the ageing peptides. Classification is performed by determining the Euclidian distance (defined as the SVM classification score) of the vector to a maximal margin hyperplane. The SVM model was optimized by the take-one-out procedure (systematic reduction of peptides in the classifier) with respect to an optimal result after cross-validation. This led to the inclusion of forty peptides in the SVM model with C and the gamma values in the SVM model of 3.2 and 0.008, respectively. This SVM model of mouse aging related urinary peptides was used to study the effect of apelin treatment.

### Urinary biochemistry

The concentration of creatinine in urine was measured by the colorimetric method of Jaffe according to the protocol Creatinine Assay Kit (Bio Assay Systems). The concentration of urinary albumin was determined by ELISA using the AlbuWell kit (Exocell).

### Renal histology and image analysis

Two-micrometer-thick cross-sections of kidney cortex were prepared and deparaffinized. The staining for Masson’s Trichrome was realized as described in. Sections were also stained for collagens by using 0.1% sirius red in water saturated picric acid. After endogenous peroxidase blockage (S2001, DakoCytomation), sections were incubated at room temperature with primary antibodies for the detection of F4/80 positive inflammatory cells (1/100, 45 min, RM2900, Caltag Laboratories) followed by anti-rabbit IgG Dako Envision HRP system (30 min, K4002, DakoCytomation). Slides were scanned using a Nanozoomer 2.0 RS (Hamamatsu Photonics SARL) and image analysis was realized in a blind fashion using Image J software (https://imagej.nih.gov/ij/). First medullar part of the slides and background was discarded. Glomerular volume was estimated from Masson Trichrome stained slides. All the glomeruli present on a slide (mean n = 180) were isolated and area was measured. Volume is estimated according to Weibel^35^ as Volume = Area^1.5^ × 1.38/1.01.

Blue staining was then quantified and expressed as percent of glomeruli area (% glomerular fibrosis), or cortex area without glomeruli (% tubular fibrosis). For fibrosis estimation in Sirius Red stained slides, the red color area was quantified in the renal cortex and expressed as percent of total cortex area. For F4/80 stained slides, the area of inflammatory cells was measured and expressed as percent of total cortex area.

### qPCR

Total RNA (1 µg) was isolated from kidney using the GeneJet RNA Purification kit (K0732, Fermentas, Thermo Scientific) and were reverse transcribed using random hexamers and Superscript II reverse transcriptase (Multiscribe, Applied Biosystem). The same reaction was performed without Superscript II (RT–) to estimate DNA contamination. Real time PCR was performed starting with 6.25 ng cDNA and both sense and antisense oligonucleotides in a final volume of 10 µl in a 384 well plate using the SYBR green universal PCR master mix (Eurogentec). Analysis of GAPDH mRNA was performed to normalize gene expression. Results are expressed as: 2^(CtGAPDH−Ctgene)^[1 − (1/2^(Ctgene−CtRT–)^)], where Ct corresponds to the number of cycles needed to generate a fluorescent signal above a predefined threshold. The primer sequences are indicated in Table 2.

**Table 2:** Primer sequences for RT-PCR analysis.

Primer	Sense	Antisense
Atg4	TTGTACCTCCAGCCAAGCC	CCTCCAGCTCAAAGTCCTCC
Atg6	CAGGATGGACGTGGAGAAAG	TTGTCCACTGCTCCTCCG
Atg12	GGACCATCCAAGGACTCATTG	AATAAACAACTGTTCCGAGGCC
P62	GAATACCTTTGCCTCCCACAC	CCTTCGTAACTCTGTTCTGCGT
LC3	GTGTCCACTCCCATCTCCG	TGCTGTCCCGAATGTCTCC
Bnip3	AGCTGCTTAAATCGGGCG	TACAGCATGTCAAGGAGGCAG
Caspase 3	AATTCAAGGGACGGGTCATG	GCTTGTGCGCGTACAGTTTC
Klotho	CCCTGTGACTTTGCTTGGG	CCCACAGATAGACATTCGGGT
APJ	GCTGTGCCTGTCATGGTGTT	CACTGGATCTTGGTGCCATTT
Apelin	GTTTGTGGAGTGCCACTG	CGAAGTTCTGGGCTTCAC
Elabela	TCATTCTCGAGTGCCCTTCC	TCCTGAGGTTGTTTTTCCGGT
IL6	GCCCACCAAGAACGATAGTCA	CAAGAAGGCAACTGGATGGAA
TNFa	TGGGACAGTGACCTGGACTGT	TTCGGAAAGCCCATTTGAGT
Collagen1	TGTGTGCGATGACGTGCAAT	GGGTCCCTCGACTCCTACA
Collagen3	AAGGCGAATTCAAGGCTGAA	TGTGTTTAGTACAGCCATCCTCTAGAA
aSMA	GTCCAAGACATCAGGGAGTAA	TCGGATACTTCAGCGTCAGAA

### Statistics

Data are expressed as the mean ± the standard error to the mean (s.e.m). Statistical analyses were performed using two-way ANOVA, to detect effect of age and effect of apelin treatment, followed by Tukey multiple comparison of means to detect interactions between age and apelin, with R software. P-values less than 0.05 (*), 0.01 (**) or 0.001 (***) were taken as statistically significant.

## Data Availability

The datasets generated during and/or analysed during the current study are available from the corresponding author on reasonable request.
